# Unilateral Idiopathic Hypertrophic Olivary Degeneration: A Case Report

**DOI:** 10.7759/cureus.85211

**Published:** 2025-06-01

**Authors:** Hasham Ramzan, Aakash Mahajan, Tarun Jain

**Affiliations:** 1 Radiology, North Canberra Hospital, Canberra, AUS; 2 Radiology, Qscan Group and Canberra Health Services, Canberra, AUS

**Keywords:** dentato-rubro-olivary tract, guillain-mollaret triangle, hod, hypertrophic olivary degeneration, mri brain

## Abstract

Hypertrophic olivary degeneration (HOD) is a rare type of trans-synaptic degeneration affecting the inferior olivary nucleus (ION), most commonly associated with lesions in the Guillain-Mollaret triangle (GMT). Because of its rarity, HOD remains poorly understood and lacks a comprehensive classification system. We present an unusual case of unilateral, idiopathic, nonlesional HOD in an 82-year-old male who exhibited gait disturbance and cognitive impairment. MRI showed hypertrophy of the right medullary olive without the typical clinical signs usually seen in HOD. This case underscores the variability in HOD presentation and highlights the need for further research into its atypical manifestations.

## Introduction

Hypertrophic olivary degeneration (HOD) is an uncommon neurological condition characterized by enlargement and T2 hyperintensity of the inferior olivary nucleus (ION) on MRI, typically resulting from trans-synaptic degeneration [1]. This process is most often triggered by disruption of the dentato-rubro-olivary pathway - also known as the Guillain-Mollaret triangle (GMT) - a neural circuit involved in motor coordination that connects the dentate nucleus, red nucleus, and ION via the superior cerebellar peduncle and central tegmental tract [2].

HOD is most frequently associated with identifiable structural lesions such as infarcts, hemorrhages, tumors, or demyelinating processes affecting this pathway [2]. Clinically, it may present with oculopalatal tremor or other cerebellar signs, although asymptomatic cases have also been reported [3]. The radiological features of HOD typically develop weeks to months after the inciting event and may persist or change over time [1].

Despite its well-characterized imaging profile, idiopathic and nonlesional forms of HOD remain poorly understood due to their rarity [4]. In this report, we describe a unique case of unilateral HOD in an elderly male patient, identified on MRI in the absence of a clear causative lesion. This case underscores the diagnostic importance of imaging in detecting atypical presentations of HOD and adds to the limited literature on idiopathic variants.

## Case presentation

**Figure 1:**
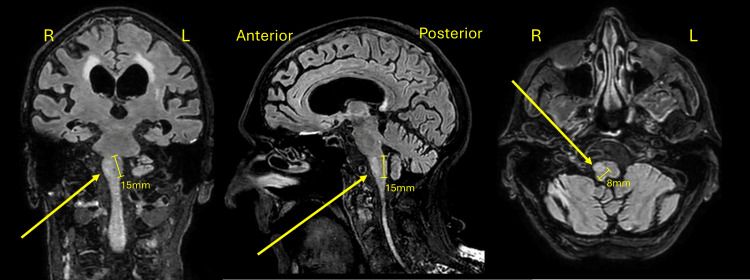
Coronal, sagittal, and axial MRI views illustrating HOD Left image: Coronal FLAIR sequence showing increased signal intensity in the right ION, indicated by the arrow, consistent with HOD. Middle image: Sagittal T2 FLAIR sequence highlighting increased signal in the right ION (arrow), corresponding to HOD. Right image: Axial FLAIR sequence displaying increased signal in the right ION, as pointed out by the arrow, indicative of HOD. FLAIR, fluid-attenuated inversion recovery; HOD, hypertrophic olivary degeneration; ION, inferior olivary nucleus

**Figure 2:**
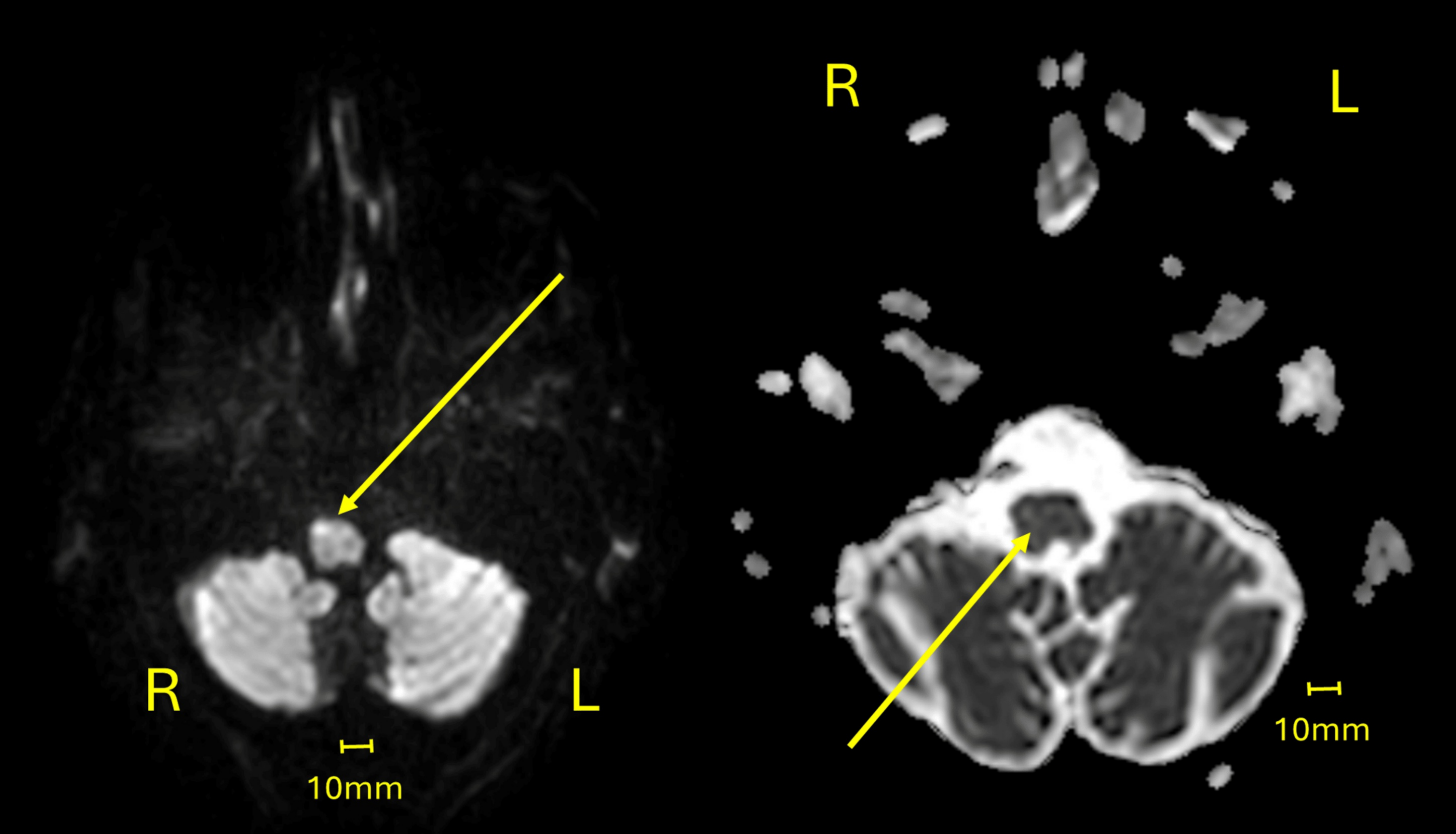
Axial DWI and ADC maps used to exclude ischemic changes Left image: DWI axial view demonstrating a subtle increase in signal intensity within the ION (arrow). Right image: ADC axial view showing no corresponding change in signal intensity in the same region (arrow). Note: Due to the nature of DWI, DWI and ADC images inherently have lower resolution and clarity, but they remain diagnostically reliable. ADC, apparent diffusion coefficient; DWI, diffusion-weighted imaging; ION, inferior olivary nucleus

An 82-year-old male with a medical history of arrhythmia and seizure disorder was admitted to a geriatric unit following recurrent falls and progressive cognitive decline. Physical examination revealed a shuffling gait, bilateral bradykinesia, left-sided cogwheel rigidity, and a right-hand intention tremor. MRI with fluid-attenuated inversion recovery (FLAIR) sequences showed increased signal intensity and subtle hypertrophy of the right ION, suggestive of HOD. No lesions were identified along the dentato-rubro-olivary pathway (GMT), and diffusion-weighted imaging (DWI) excluded acute ischemia and other mimicking pathologies. These findings supported a diagnosis of nonlesional, idiopathic HOD (Figure 1, Figure 2).

The treating team did not specifically address the finding of HOD, instead focusing on the broader clinical picture that supported a diagnosis of Parkinson-plus syndrome (PPS). This conclusion was based on the patient’s clinical features, characteristic imaging findings, and a limited response to a levodopa trial. The patient showed moderate improvement with levodopa and physiotherapy and was subsequently discharged to a residential aged care facility. At the time of discharge, no further neurological deterioration had been reported.

## Discussion

While the pathophysiology of HOD is well described, its clinical and radiological presentations remain highly variable [1,4]. In most cases, HOD is secondary to identifiable lesions affecting the dentato-rubro-olivary pathway, but idiopathic and nonlesional forms, such as the one presented here, are exceedingly rare [1,4]. The imaging findings in our patient, including T2/FLAIR hyperintensity and subtle hypertrophy of the right ION, were consistent with HOD, despite the absence of any causative lesion on MRI.

This case highlights the diagnostic value of MRI in detecting HOD even without classical symptoms or a known precipitating event. DWI was essential in ruling out acute ischemia, while the absence of contrast enhancement further supported a noninflammatory, nonneoplastic etiology. Although the patient’s broader clinical picture suggested a neurodegenerative process, the HOD findings appear to be an independent radiological observation rather than a direct manifestation of the underlying syndrome.

Palatal tremor is the most recognized symptom of HOD, occurring in roughly one-third of cases [5]. Other manifestations such as Holmes tremor, ocular myoclonus, and ataxia have also been reported [5]. However, HOD can be discovered incidentally during imaging for unrelated neurological issues [6]. In our patient, the lack of classical symptoms such as palatal tremor, combined with the absence of a causative lesion, underscores the diagnostic challenge and the importance of radiological recognition.

Although the clinical presentation was consistent with a PPS, the presence of HOD should not be assumed to be directly caused by PPS. While some case reports suggest a possible association between the two conditions, larger, methodologically robust studies have not demonstrated a statistically significant link [7]. Given the rarity of co-occurrence of both PPS and HOD, it remains difficult to draw definitive conclusions. Some evidence hints at a potential relationship, but current data are insufficient to establish causality [7].

This case contributes to the limited literature on HOD, particularly the rare subset of unilateral, idiopathic, nonlesional presentations. While HOD is traditionally associated with lesions in the dentato-rubro-olivary pathway, recent analyses suggest that a significant proportion of cases - up to 50% in some series - may lack identifiable structural lesions [4]. However, truly idiopathic and unilateral cases remain exceedingly rare, with most documented instances being bilateral or secondary to a known insult [4]. By reporting this case, we support the growing recognition of nonclassical HOD presentations and emphasize the need for continued documentation to better define its clinical spectrum and diagnostic criteria.

## Conclusions

This case highlights the diagnostic nuance required when interpreting medullary signal abnormalities, especially in patients with overlapping neurodegenerative features. While HOD is often associated with structural lesions or classical symptoms, this report illustrates that it can also present in a nonlesional, idiopathic form with atypical clinical features.

From a research perspective, this case adds to the growing recognition of HOD’s heterogeneity and supports the need for prospective studies to better define its natural history, clinical significance, and potential associations with other neurodegenerative disorders such as PPS. As imaging technology and diagnostic criteria evolve, clearer classification of HOD subtypes may improve diagnostic accuracy and patient management.

## References

[1] Onen MR, Moore K, Cikla U, Ucer M, Schmidt B, Field AS, Baskaya MK (2018). Hypertrophic olivary degeneration: neurosurgical perspective and literature review. World Neurosurg.

[2] Ogut E, Armagan K, Tufekci D (2023). The Guillain-Mollaret triangle: a key player in motor coordination and control with implications for neurological disorders. Neurosurg Rev.

[3] Shaikh AG, Hong S, Liao K (2010). Oculopalatal tremor explained by a model of inferior olivary hypertrophy and cerebellar plasticity. Brain.

[4] Gu CN, Carr CM, Kaufmann TJ, Kotsenas AL, Hunt CH, Wood CP (2015). MRI findings in nonlesional hypertrophic olivary degeneration. J Neuroimaging.

[5] Bhattacharjee S (2020). Palatal tremor - pathophysiology, clinical features, investigations, management and future challenges. Tremor Other Hyperkinet Mov (N Y).

[6] Gao Q, Li Z, Guo C (2022). Hypertrophic olivary degeneration: a description of four cases of and a literature analysis. Quant Imaging Med Surg.

[7] Hanihara T, Amano N, Takahashi T, Itoh Y, Yagishita S (1998). Hypertrophy of the inferior olivary nucleus in patients with progressive supranuclear palsy. Eur Neurol.

